# Non-invasive *In Vivo* Imaging of Cancer Using Surface-Enhanced Spatially Offset Raman Spectroscopy (SESORS)

**DOI:** 10.7150/thno.36321

**Published:** 2019-08-13

**Authors:** Fay Nicolson, Bohdan Andreiuk, Chrysafis Andreou, Hsiao-Ting Hsu, Scott Rudder, Moritz F. Kircher

**Affiliations:** 1 Department of Radiology, Memorial Sloan Kettering Cancer Center, New York, New York 10065, United States; 2 Department of Electrical and Computer Engineering, University of Cyprus, Nicosia, Cyprus; 3 Innovative Photonic Solutions, Monmouth Junction, New Jersey 08852, United States; 4 Center for Molecular Imaging and Nanotechnology (CMINT), Memorial Sloan Kettering Cancer Center, New York, New York 10065, United States; 5 Molecular Pharmacology Program, Sloan-Kettering Institute, New York, New York 10065, United States; 6 Department of Imaging, Dana-Farber Cancer Institute and Harvard Medical School, Boston, Massachusetts 02215, United States

**Keywords:** cancer imaging, glioblastoma, in vivo, nanoparticles, Raman, SERS, SERRS, SORS, SESORS, SESORRS, optical imaging, spectroscopy.

## Abstract

**Rationale**: The goal of imaging tumors at depth with high sensitivity and specificity represents a significant challenge in the field of biomedical optical imaging. 'Surface enhanced Raman scattering' (SERS) nanoparticles (NPs) have been employed as image contrast agents and can be used to specifically target cells *in vivo.* By tracking their unique “fingerprint” spectra, it becomes possible to determine their precise location. However, while the detection of SERS NPs is very sensitive and specific, conventional Raman spectroscopy imaging devices are limited in their inability to probe through tissue depths of more than a few millimetres, due to scattering and absorption of photons by biological tissues. Here, we combine the use of "Spatially Offset Raman spectroscopy" (SORS) with that of "surface-enhanced resonance Raman spectroscopy" (SERRS) in a technique known as "surface enhanced spatially offset resonance Raman spectroscopy" (SESO(R)RS) to image deep-seated glioblastoma multiforme (GBM) tumors *in vivo* in mice through the intact skull.

**Methods**: A SORS imaging system was built in-house. Proof of concept SORS imaging was achieved using a PTFE-skull-tissue phantom. Imaging of GBMs in the RCAS-PDGF/N-tva transgenic mouse model was achieved through the use of gold nanostars functionalized with a resonant Raman reporter to create SERRS nanostars. These were then encapsulated in a thin silica shell and functionalized with a cyclic-RGDyK peptide to yield integrin-targeting SERRS nanostars. Non-invasive *in vivo* SORS image acquisition of the integrin-targeted nanostars was then performed in living mice under general anesthesia. Conventional non-SORS imaging was used as a direct comparison.

**Results**: Using a low power density laser, GBMs were imaged via SESORRS in mice (n = 5) and confirmed using MRI and histopathology. The results demonstrate that via utilization of the SORS approach, it is possible to acquire clear and distinct Raman spectra from deep-seated GBMs in mice *in vivo* through the skull. SESORRS images generated using classical least squares outlined the tumors with high precision as confirmed via MRI and histology. Unlike SESORRS, conventional Raman imaging of the same areas did not provide a clear delineation of the tumor.

**Conclusion**: To the best of our knowledge this is the first report of *in vivo* SESO(R)RS imaging. In a relevant brain tumor mouse model we demonstrate that this technique can overcome the limitations of conventional Raman imaging with regards to penetration depth. This work therefore represents a significant step forward in the potential clinical translation of SERRS nanoparticles for high precision cancer imaging.

## Introduction

Despite notable advances in the detection and treatment of cancer, high grade gliomas such as glioblastoma multiforme (GBM = WHO grade IV astrocytoma) represent one of the greatest challenges in medicine 1. Even with the most advanced treatment regimen, GBMs are associated with a median survival time of only approximately 15 months 2. Common symptoms include progressive focal neurological deficits, headaches and seizures, however, due to the aggressive nature of GBMs, they often evade incidental discovery. Usually, the initial diagnosis is based on suspicious findings on magnetic resonance imaging (MRI) 2, followed by biopsy. Contrast-enhanced computed tomography (CT) can be used as an alternative, however it suffers from decreased soft-tissue contrast 2, 3. Both MRI and CT imaging systems are associated with significant costs and are limited in their specificity, thus relying on the need for biopsies to provide cellular and molecular information. While theranostic approaches to improve diagnosis and cure rates of various cancer types is a very active area of research with multiple approaches being tested 4-18, complete tumor resection, which can only be achieved via complete tumor visualization, is deemed the most important factor for the outcome of GBM patients.

Relying on the inelastic scattering of light, Raman spectroscopy is a nondestructive method that provides information on the molecular composition and structure of a sample 19. Its applicability in its intrinsic form, i.e. without the use of any amplification strategy, has been demonstrated extensively in a number of applications including the characterization of *ex vivo* biopsy samples 19, and, in a few limited examples, *in vivo* imaging 19-21. Whilst such intrinsic approach has shown promise in being able to discriminate between the different tissue types, it is often associated with poor signal to noise ratios and long acquisition times, which represent major hurdles for *in vivo* applications 22. Therefore, nanoparticle (NP) based contrast agents have been developed that cause amplification of the signal intensity via the "surface-enhanced Raman spectroscopy" (SERS) phenomenon 23. By utilizing the plasmonic effects of gold nanoparticles to enhance the Raman signal of a dye molecule adsorbed onto their surface, SERS has been shown to yield enhancements of several orders of magnitude greater than intrinsic Raman spectroscopy 23. Moreover, through the use of a Raman reporter which has an electronic transition that corresponds to that of the laser excitation wavelength, 'surface-enhanced *resonance* Raman spectroscopy' (SE*R*RS) has been shown to generate even further enhancements in Raman signal 24, 25. Due to the unique Raman “fingerprint” spectroscopic signature, SERS nanotags can be tracked with extraordinary specificity, which is one of the major advantages of SERS over fluorescence imaging 26. In addition, targeted SERS contrast agents, i.e. SERS NPs functionalized with biomolecules such as antibodies 27 or DNA aptamers 28, have been explored for the active targeting and detection of cancer *in vivo* using Raman spectroscopy. However, despite several reports in the literature showing the potential to target and image tumors *in vivo* using SERS, these studies typically rely on xenograft models where the cancerous cells are injected subcutaneously, with the tumors therefore located at very shallow depths 29. Therefore, superficial Raman imaging of tumors using SERS is not representative of the clinically much more common scenario, where tumors are located at greater depth within the body. The inability to image at such increased depth is a major limitation for conventional Raman spectroscopy systems that collect the Raman photons through the same path as that of the excitation laser. Currently, the majority of commercially available Raman spectrometers are based on such conventional spectroscopy systems. Such approach is limited with regards to depth penetration of the incident light in diffusely scattering media such as tissue beyond a few millimeters, thus making it extremely difficult to detect tumors at clinically relevant depths 30, 31.

First reported by Matousek *et al*., 'spatially offset Raman spectroscopy' (SORS) aims to overcome the depth limitations associated with conventional Raman spectroscopy 32. SORS is a relatively new technique which is still being explored and refined by several groups. Unlike other optical techniques such as fluorescence, Raman does not rely on absorption processes but rather the inelastic scattering of light. As such, SORS utilizes the concept of random scattering processes in which deeper born Raman photons are less likely to migrate back to the point of incidence 33. Thus, following interaction with incident light, photons emerging from deeper layers in turbid media have to traverse larger distances via diffuse migration compared to photons generated at shallower depths 30, 34. Using a SORS approach, it is therefore possible to collect photons from deeper subsurface regions by offsetting the point of laser excitation from the collection optics. In recent years the introduction of SORS has also opened up new avenues for medical applications, namely non-invasive disease diagnostics. Examples include the analysis of bone composition 35-37, and cancerous tissue 38-41.

'Surface-enhanced spatially offset Raman spectroscopy' (SESORS) combines the depth penetration benefits of SORS with the signal enhancing capabilities of SERS to achieve improved sample interrogation at greater depths 42. Using a transmission (180º offset) geometry, bisphosphonates have been detected *ex vivo* in bone through 2 cm of tissue 43, and Stone *et al*. successfully detected SERS nanotags embedded in a tissue phantom at depths of up to 5 cm 42, 44. In other SESORS studies where, unlike transmission Raman, the collection optics are on the same side as the incident beam, although still spatially offset from each other, SERS nanotags have been detected through 6.75 mm of tissue 45, and through 8 mm of bone 46. Sharma *et al*. successfully demonstrated the potential to detect neurotransmitters at concentrations as low as 100 µM in a brain tissue phantom through a cat skull 47 and more recently, using an inverse SORS approach, Vo-Dinh *et al*. demonstrated the potential to recover SERS spectra, albeit not images, through a monkey skull 48. In this instance gold nanostars were suspended in an agarose gel phantom and shielded by a monkey skull with a thickness of 5 mm. The detection of *ex vivo* multicellular tumor spheroids has also been reported using the SESORS technique through depths of 15 mm of tissue using a handheld SORS spectrometer 49, 50.

However, whilst several reports have explored the use of SERS nanotags for *in vivo* cancer imaging using conventional Raman spectroscopic methods, or the use of SESORS to probe through more clinically relevant depths *ex vivo*, SESORS imaging has not yet been reported* in vivo*. Here, we report - for the first time - the imaging of targeted SERS nanotags using SESORS *in vivo* in live and intact mice. This was achieved using a highly Raman active SERRS nanotag functionalized with a cyclic RGD peptide to successfully target and image GBMs *in vivo* by targeting integrin receptors such as α_v_β_3_ which are overexpressed on the tumor vasculature, angiogenic endothelial cells and tumor cells. Importantly, the state-of-the-art RCAS-PDGF/N-tva transgenic mouse model of GBM used in this study presents with all the histopathological and imaging hallmarks of human high-grade tumors such as infiltrating tumor, margins, microvascular proliferation and pseudopalisading necrosis 51, 52. Previous work in our group has demonstrated the successful application of SERRS active nanotags to target and image microscopically cells overexpressing integrin α_v_β_3_ in a transgenic mouse model of GBM, however this was achieved *ex vivo*, i.e. the mice were sacrificed and the brain was then removed from the skull and imaged using confocal Raman microscopy 53. By advancing the optical approach through which the Raman photons are collected by means of SORS, here we have successfully imaged gliomas *in vivo* without the need to rely on *ex vivo* Raman analysis techniques. Moreover, unlike previous work where subcutaneous xenograft models were used or precise brain tumor delineation necessitated craniotomy 54, we report the successful detection of SERS nanotags through not only tissue, but also the skull. As such, to the best of our knowledge, this is not only the first report of specific targeting and detection of a brain tumor *in vivo* using SESORS but also the first general report of the detection of SERS nanotags *in vivo* using a SORS approach.

## Materials and Methods

### Reagents

All chemicals were purchased from Sigma-Aldrich and used as received unless otherwise stated. Paraformaldehyde (16%) was purchased from Thermofisher and diluted to 4% in phosphate buffered saline (pH 7.1). Cyclic RGDyK peptide was purchased from Biomatik and used as received. DI water (18 MΩ⋅cm) was used in all experiments.

### SERRS nanotag synthesis

The synthesis of SERRS active gold nanostars was performed according to recent protocols published by our group, with minor modifications as detailed below 55, 56.

### Synthesis of gold seeds

The synthesis of 5 nm gold seeds was carried out in accordance with a previous report 57. Two ml of 25 mM solution of HAuCl_4_ were added to 200 ml of DI water. Following this, 6 ml of freshly-prepared ice-cold 100 mM NaBH_4_ solution was added to the mixture under stirring. After 30 seconds the mixture was diluted 5 times with DI water and left overnight at room temperature before usage. A TEM image of the gold seeds can be found in **Figure S1** (Supporting Information).

### Synthesis of bare gold nanostars

The seed-mediated synthesis of gold nanostars was performed as follows: in a cold room (+4 ºC) 800 ml of 200 mM solution of ascorbic acid was cooled to 0 ºC using a salt-ice bath. Under turbulent stirring, 7 ml of 20 nM gold seeds were added to the mixture, followed by 40 ml of 5 mM solution of HAuCl_4_. After 10 seconds a color change was observed from colorless to deep blue. The resulting deep blue reaction mixture was transferred to 50 ml Falcon tubes and centrifuged at 0 ºC and 3320 × *g* for 20 mins. Following centrifugation, the transparent supernatant was discarded and the liquid pellet on the bottom of the tubes was collected. All 16 pellets were combined in a single 3 ml Slide-A-Lyser G2 dialysis cassette with 3.5k MWCO and subjected to dialysis versus DI water for the three days. The DI water was changed daily over the 72 hour period.

### Silication and Raman reporter dye attachment

To a 50 ml falcon tube (tube A), 0.55 ml of tetraethyl orthosilicate, 20 ml of isopropanol, 0.08 ml of 20 mM IR 792 dye solution in DMF, and 0.3 ml of DI water were added together. To a 15 ml falcon tube (tube B), 2 ml of 0.2 nM bare nanostars and 6 ml of ethanol were added. Immediately before combining tube A and tube B, 0.4 ml of ammonium hydroxide solution (28% aq) was added to tube B. Tube A was put on a vortex mixer and the contents of tube B were added rapidly under turbulent mixing. The resulting mixture was left on a shaker for 20 min at room temperature. Afterwards, tube A was filled to 50 ml with ethanol and centrifuged at 3320 × *g* at 0 ºC for 20 min. Green supernatant was discarded, leaving ~0.5 ml of solution with a dark blue liquid pellet on the bottom of the tube. The pellet was sonicated to fully homogenize the solution and transferred to a 1.5 ml Eppendorf tube, which was then filled with ethanol. The tube was centrifuged at 8000 × *g* at room temperature for 4 minutes and the supernatant discarded. The resulting pellet was then resuspended in ethanol and subjected to sonication. The ethanol washing process was repeated four more times. This was followed by two further washing steps with DI water to complete the process. The resulting aqueous solution can be stored in a fridge for up to one week.

### Surface modification with thiol groups

Aqueous solutions of nanostars were centrifuged in 1.5 ml LoBind Eppendorf tubes at 8000 × *g* at room temperature for 4 minutes. The aqueous supernatant was then discarded, leaving the pellet and some supernatant, (total volume of 0.1 ml). Next, 0.9 ml of ethanol was added and the mixture was sonicated. Subsequently, 0.1 ml (3-mercaptopropyl)-trimethoxysilane was added and the mixture was left on a shaker for 1h at room temperature. Then it was centrifuged at 8000 × *g* at room temperature for 4 minutes, twice washed with ethanol, washed with water and finally the pellet was resuspended in 10 mM HEPES buffer pH 7.2.

### Surface modification with targeting RGD peptide

6.2 mg of cRGDyK peptide and 40 mg of Mal-PEG4000-NHS (CAS 851040-94-3) were dissolved in 10 ml of 10 mM HEPES buffer pH 7.2 and left on a shaker overnight to produce 1 mM solution of Mal-PEG4000-cRGDyK conjugate. 0.04 ml of this solution was added to a 1 ml (0.4 nM) solution of thiolated nanostars in 10 mM HEPES buffer pH 7.2 in a 100,000:1 PEG/NPs ratio and shaken for 2h at room temperature. 1 µl of hydroxylamine (50% aq) was added to the solution and left for 10 min to quench any unreacted NHS. The nanoparticles were spun down and washed 3 times with 10 mM HEPES buffer pH 7.2. This solution of nanoparticles remained stable in the fridge for up to 24 hours. Prior to injection, the nanoparticles were concentrated to 8 nM by spinning down 2 ml of solution (8000 × *g*, 4 mins) and resuspending the pellet in 100 µL of the remaining supernatant.

### Integrin-targeted SERRS nanoparticle characterization

Transmission electron microscopy (TEM) was performed on a JEOL JEM 1400 Transmission Electron Microscope at 100 kV. The sample was prepared as follows: 5 µL of ~0.1 nM nanoparticles solution was left to adsorb onto a PEI-covered TEM grid (CF300-Cu, Electron Microscopy Sciences) for 30 min. The remaining solution was washed off with water and the dry grids were used for imaging. Zeta potential and hydrodynamic size measurements were performed using Zetasizer Nano ZS (Malvern) on particles dispersed in 10 mM HEPES buffer (pH 7.2), (Table S1, Supporting Information). Nanoparticle tracking analysis (NTA) NS500 (Malvern) was used to determine nanoparticle concentration. The concentration of gold seeds was calculated from the absorption spectrum. SpectraMax ID 5 plate reader (Molecular Devices) was used for optical characterization of nanoparticles (**Figure S2**).

### Animal Models

All animal experiments were approved by the Institutional Animal Care and Use Committees of Memorial Sloan Kettering Cancer Center (#06-09-013). The somatic gene transfer system RCAS/tv-a was used to generate the glioblastoma-bearing mice 51, 52, 58. Briefly, the 4-8 week-old *Nestin*-tv-a/*Ink4a-arf*-/-/*Pten^f^*^l^/^fl^ mice were stereotactically injected with 1×10^5^ DF-1 cells transfected with RCAS-*Pdgfb* and RCAS-Cre (1:1 mixture, 1 µL) into the brain, coordinates bregma 2 mm (anterior), 2 mm (left), and depth 2.5 mm from the surface. The genetic aberrations including overexpression of oncogene Pdgfb and loss of tumor suppressor genes (I*nk4a*-*arf* and *Pten*) led to near complete penetrance of glioblastoma within 4 weeks. Tumor incidence and size were determined by weekly MRI scans 4.7 T animal MRI (Bruker Biospin, Billerica, MA) starting four weeks after DF-1 cell injection.

### Custom-built SORS Imaging System

**Figure 1:**
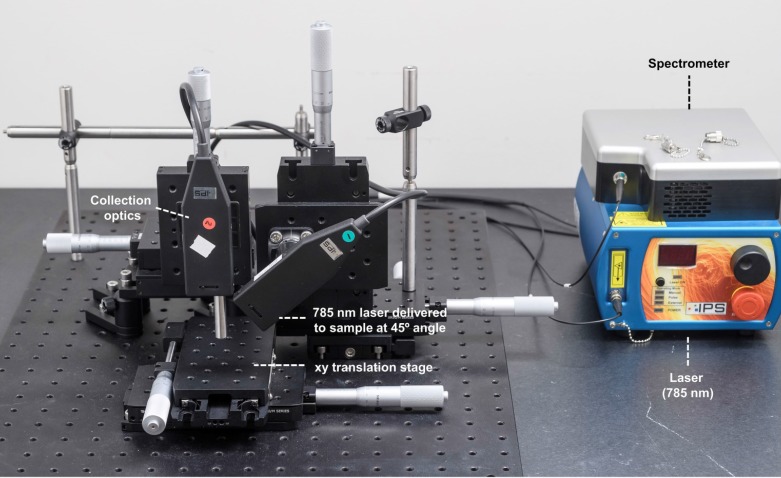
** SORS setup.** A 785 nm laser was delivered at a 45˚ angle with regards to the collection optics. A translational xyz stage was used to move the laser away from the point of collection in order to apply the SORS technique.

All SORS measurements were carried out using an in-house built system, the design of which was based on previous reports in the literature 45, 59, 60. A 785 nm laser (Innovative Photonics Solutions) was coupled to one of two fiber optic probes (Innovative Photonics Solutions). The lens was removed from the probe connected to the laser to deliver a diffuse beam to the sample. Both probes were mounted on individual *xyz* translational stages (Thorlabs) and a rotation mount was used to deliver the excitation light to the sample at a 45˚ angle. The incident beam on the sample surface was therefore elliptical, with the shorter radius being 1.5 mm and the longer 2 mm. The collection probe was mounted at a 45˚ angle with regards to the excitation probe, i.e. at 180˚ angle from the sample surface. The collection probe (0.22 NA, 9.7 mm working distance) was coupled to a high throughput f/2 spectrometer (Innovative Photonics Solutions), to collect the scattered Raman photons. It was estimated that the diameter of the Raman collection region on the sample surface was 200 µm. The SORS setup is shown in **Figure 1**. The zero offset was ascertained by delivering the angled excitation beam (probe 1) onto the surface of PTFE. The excitation beam was then moved in the x-direction towards the collection probe (probe 2) until maximum contribution from PTFE was observed in the acquired spectra. Probe 2 was then focused in the z-direction. This approach ensures that the collection spot area substantially overlaps the excitation area at zero offset. The spatial offset was controlled by translating laser beam away from the focal point of the collection probe in the region of a few mm (Δx). This directed the beam at an appropriate point on the sample, e.g. plastic or mouse. All samples were positioned on a third translational *xy* stage (Thorlabs) to allow freedom to move the sample without impacting the alignment between the excitation and collection probes.

### SORS Imaging

Acquisition times were varied depending on the sample under study. Control spectra were generated by coupling the collection probe to the laser in order to excite and collect through the same probe. Laser power varied depending on the sample under study. Laser output was measured with a handheld laser power meter (Edmund Optics, Inc.). For SORS measurements using plastic and tissue phantoms, 400 mW powers were used. For SORS measurements using animal models, the laser power was lowered to approximately 130 mW at the sample surface.

### Conventional Raman Imaging

Conventional (non-SORS) Raman (CR) imaging was used as a control in order to carry out a direct comparison between CR or SORS approaches. The SORS system described above was therefore modified so that the laser excitation and Raman photon collection occurred through the same lens, i.e. without the use of a spatial offset. For control measurements, the focused spot size was roughly 500 µm in diameter, resulting in a higher power density. The laser power used for CR imaging was 20 mW. All laser spot sizes were determined using a beam profiler (Dual Scanning Slit Beam Profiler, 200 - 1100 nm, Thorlabs).

### SORS optimization measurements

Both polytetrafluoroethylene (PTFE) and pink polypropylene (PP) sheets were purchased from a plastic retailer and cut up into smaller pieces. PTFE (length 5 cm × width 2 cm × thickness 2 mm) was placed on the stage and varying numbers of PP sheets (length 5 cm × width 2 cm × thickness 2 mm) were then placed on top of the PTFE to act as a barrier. The thickness of the barrier was increased by adding further PP sheets. SORS mapping of PTFE through the skull was carried out by gluing (Gorilla glue) a small piece of PTFE underneath the skull of a sacrificed 8 week old mouse. The skull was then filled with 1% agarose gel to create a phantom 47. This phantom was then wrapped in thin slices of pork to create a GBM analogue.

### *In vivo* imaging using SESORS

Tumor bearing mice were anaesthetized using isoflurane (2%) inhalation. Mice were shaved using an electric shaver and residual hair removed using hair removal cream. Each mouse was placed onto the *xy* translational stage with a range of 50 mm in each direction and moved in 1 mm increments to acquire pointwise spectra. The mice remained alive for all mapping experiments. After imaging, the mice were sacrificed by CO_2_ asphyxiation and their organs harvested for *ex vivo* analysis (**Figure S3**).

### Histology

Intact GBM-bearing brains were fixed in 4% paraformaldehyde (PFA) and then embedded in paraffin. Each brain was sliced in the same orientation as the MRI and 5 μm-thick sections were stained with hematoxylin and eosin (H&E). H&E slides were digitally scanned using the Pannoramic SCAN slide scanner (3DHistech) with a 20× objective. Images were viewed and processed using CaseViewer (3DHISTECH, Budapest, Hungary).

### Data processing

All spectra were processed using Matlab software (version 2018b, The MathWorks, Natick, MA, USA) and PLS_toolbox (Eigenvector Research). Processing of spectra for stacked graphs involved truncating and baselining the spectra as coupled with Savitzky-Golay smoothing. For the creation of false color 2D heat maps, spectra were truncated, baselined and smoothed using Savitzky-Golay filtering before the intensity of specific peaks of interest were plotted as a combination surface/contour false color 2D heat map.

### CLS

A constraint least squares (CLS) model was developed using reference spectra obtained from the nanoparticles, bone (from the skull), and tissue background (from the lower dorsal head area). All spectra (references and samples) were subjected to baseline subtraction using the Whittaker filter (λ=200). The reference spectra were normalized to unit area, whereas the samples were kept at natural units (intensity) 61. The pixel-wise scores of the data were calculated using a non-negative least square algorithm to produce the figures on the three channels (NPs, bone, background).

## Results and Discussion

**Figure 2:**
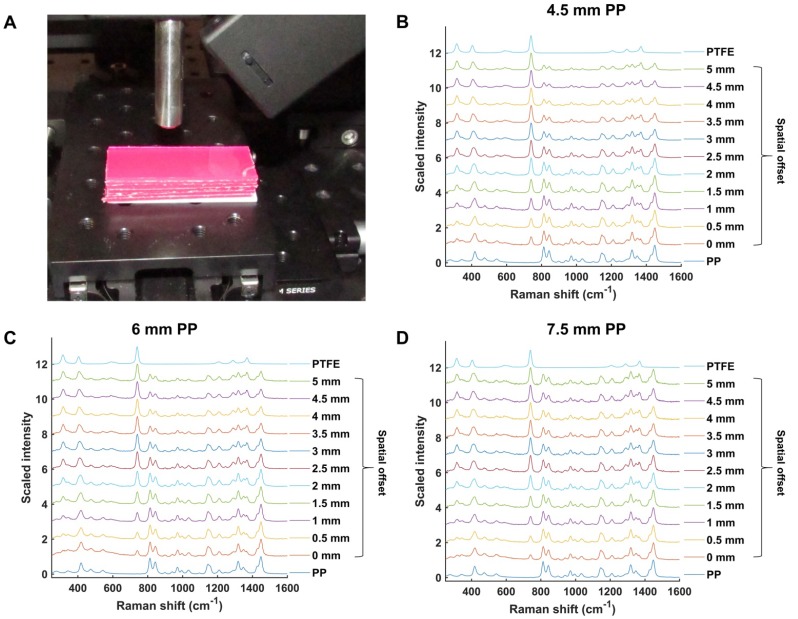
(a) Experimental setup involving PP sheets placed upon a sheet of PTFE. (b) Scaled, stacked SORS spectra of PTFE through PP with thicknesses of (b) 4.5 mm (c) 6 mm, and (d) 7.5 mm. The spatial offset was increased from 0 to 5 mm in 0.5 mm increments and the offset spectra recorded. PTFE and PP reference spectra are shown at the top and bottom, respectively. In each graph (b-d), as the spatial offset increases, contribution of PTFE (739 cm^-1^) to the acquired spectra increases. Similarly, as the spatial offset increases, the spectral contribution of PP (814 cm^-1^) to the acquired spectra decreases. All measurements were carried out using a 1 s integration time, 5 accumulations, and 785 nm laser excitation wavelength.

Initial experiments were performed in order to validate and optimize the SORS system (**Figure 1**) for through-barrier measurements. Sheets of pink polypropylene (PP) were placed on top of PTFE to create a barrier with the aim of detecting the PTFE analyte through the PP barrier. The experimental setup is shown in **Figure 2a**. Spectra of PTFE through thicknesses of 4.5, 6 and 7.5 mm of PP were collected at spatial offsets of 0 to 5 mm (0.5 mm increments). As the spatial offset increases, the spectral contribution of PTFE greatly increases (**Figure 2b-d)**. Furthermore, the relative contribution of PP to the obtained spectra decreases, thus indicating that through-barrier detection is taking place. In addition, the data clearly show that as the thickness of the PP barrier is increased, larger spatial offsets are required to obtain a significant spectral contribution from PTFE. Thus, by increasing the spatial offset, i.e. the distance of the excitation spot from the point of collection, it is possible to obtain a clear spectral contribution of PTFE through the PP barrier. This therefore provides validation that the assembled system allows for the collection of spectral information using the SORS technique to detect spectral signatures from a subsurface layer.

**Figure 3:**
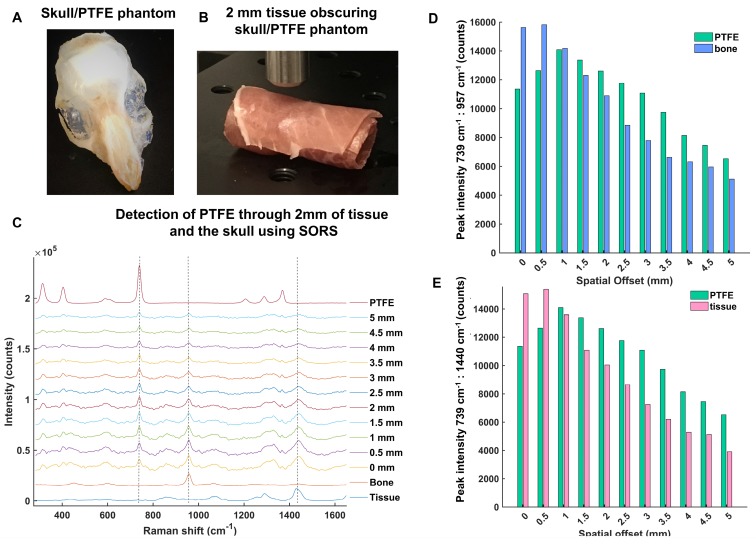
(a) Skull/PTFE phantom. The PTFE was glued underneath a mouse skull to keep it in place. The skull was then filled with agarose gel (1%) to create a brain tumor phantom. (b) Skull/PTFE phantom wrapped in 2 mm of tissue to create a phantom for the imaging of GBM through the skull. c) SORS spectra collected at varying spatial offsets (0 - 5 mm) through 2mm of tissue. (d) Peak intensities of PTFE (739 cm^-1^) in comparison to bone (957 cm^-1^) at varying spatial offsets (0-5 mm). (e) Peak intensities of PTFE (739 cm^-1^) in comparison to tissue (1440 cm^-1^) at varying spatial offsets (0-5 mm). (d) and (e) show the peak intensity at each respective wavenumber. As the spatial offset increases, the contribution of both bone and tissue to the acquired spectra decreases. Further, as spatial offset increases, the contribution of PTFE to the spectrum increases. All measurements were performed using a 785 nm laser, 2 s integration time, 5 acquisitions.

In order to develop the SORS system for *in vivo* imaging of GBM using SESORS, skull-tissue phantoms were first created. The skull from a sacrificed 8 week-old mouse was removed, cleaned and fixed in 4% PFA and a small piece of PTFE (5 mm × 5 mm, thickness 2 mm) was glued directly underneath the top of the skull (**Figure 3a**). There was no space between the PTFE and the inside surface of the skull, and the PTFE was directly fixed in position. The skull was then filled with 1% agarose gel. The PTFE was held in position by a very thin layer of glue in order to prevent it from moving following the uncontrollable evaporation of agarose gel. No spectral contribution from the glue was observed through the skull, i.e. the glue did not contribute to the acquired SORS spectra. Overall, this created a phantom, with the PTFE acting as a GBM tumor mimic. Following this, the brain-skull mimic was wrapped fully in sections of ham in order to mimic the skin (**Figure 3b)**. Porcine tissue was chosen as an analogue to human samples because its ability to mimic human tissue is greater than that from avian species 42. It is reasoned that the incorporation of agarose and wrapping of the tissue mimic fully around the skull helped to facilitate the return of scattered Raman photons to the collection optics 62.

The optimum offset for the detection of PTFE through the skull and varying thicknesses of tissue was then investigated to determine a suitable offset for SESORS imaging of GBM tumors through the skull. **Figure 3c** shows the detection of PTFE through the skull and 2 mm of tissue at spatial offsets (0 - 5 mm, 0.5 mm increments). It was determined that the thickness of the skin on top of a living mouse does not exceed 2 mm. The results demonstrate that as the excitation probe was moved further away from the point of collection, i.e. by applying the SORS approach, the contribution of the tissue barrier to the overall spectrum decreases. At spatial offsets beyond 5 mm, spectra were associated with very weak Raman scattering and poor signal-to-noise ratios, meaning they were unsuitable for providing any chemical information relating to the tumor phantom. The peak intensities of PTFE (739 cm^-1^) to bone (957 cm^-1^) and PFTE to tissue (1440 cm^-1^) are shown in **Figure 3 d and e**, respectively. The bar chart is representative of the actual Raman intensity values used to produce the scaled graph (**Figure 3c**). Spectra were obtained using a 2 s integration time, with 5 acquisitions in order to improve signal-to-noise ratios. The bar charts demonstrate that at offsets of 1.5 mm and beyond, PTFE generates a stronger relative contribution to the acquired spectra, in comparison to contributions from bone and tissue which diminish as the spatial offset is increased. Thus, despite the results demonstrating that several spatial offsets would be appropriate for probing though both the skin and skull, it was deemed that spatial offsets in the region of 2 - 3 mm would be most suitable for application of SESORS to image GBM *in vivo*.

**Figure 4:**
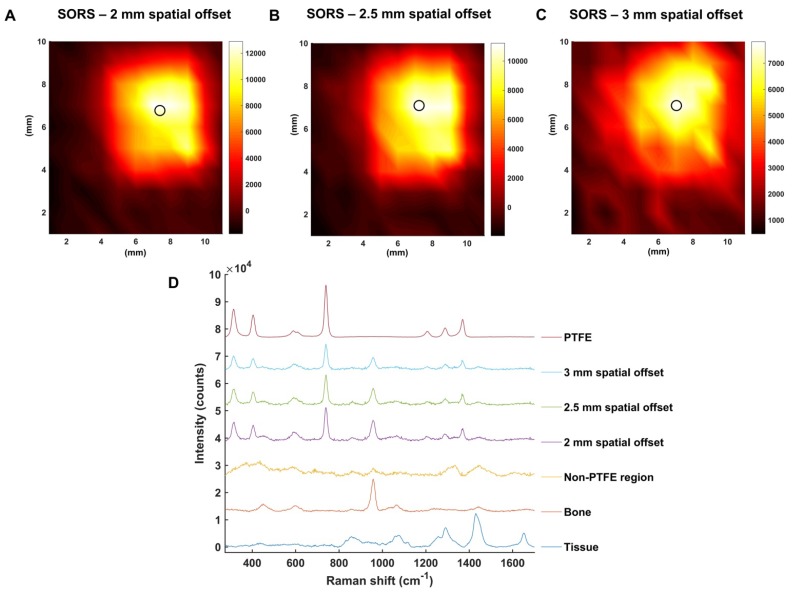
SORS false color 2D heat maps of the peak intensity at 739 cm^-1^ through 2 mm of tissue and the skull at spatial offsets of (a) 2 mm, (b) 2.5 mm and (c) 3 mm. Measurements were carried out by moving an xy translational stage in steps of 1 mm to create an image of 10 x 11 pixels. The 2D heat maps show the detection of the PTFE square which was glued underneath the skull. Each circle on the heat map refers to the area of maximum intensity generated at 739 cm^-1^. (d) Corresponding spectra collected at the point of maximum intensity at spatial offsets of 2, 2.5 and 3 mm are shown. A spectrum collected in a region where PTFE is not present is also shown and displays similar characteristics to that of tissue. This spectrum is representative for all spectra collected where the PTFE was not present. The bone and tissue reference spectra are displayed at the bottom of the stacked graph. A reference spectrum for PTFE is displayed at the top. All measurements were carried out using a 785 nm laser, 2 s integration time, and 5 acquisitions.

To investigate this further, the mapping of PTFE through 2 mm of tissue at offsets of 2, 2.5 and 3 mm was carried out (**Figure 4a, b, c,** respectively). The offset was adjusted by moving the excitation probe away from the point of collection using an additional *xyz* translational stage. A translational *xy* stage with a range of 50 mm was used to manoeuvre the tissue samples in steps of 1 mm to create an image of 10 × 11 pixels. False color 2D SORS heat maps of the peak intensity at 739 cm^-1^ were then constructed using spatial offsets of 2, 2.5 and 3 mm (**Figure 4 a-c**, respectively). **Figure 4d** displays spectra collected at the point of maximum intensity, i.e. where the PTFE was most clearly observed using SORS at spatial offsets of 2, 2.5 and 3 mm. Spectra collected in regions where the PTFE was not present is also displayed as an average spectrum (“non-PTFE region”) and consists of spectra collected at the point of minimum intensity in each heat map. Reference spectra of the bone and tissue (bottom) and PTFE (top) are also displayed for clarity.

All three heat maps demonstrate the successful detection of PTFE through 2 mm of tissue and the skull. Moreover, the area of intensity at 739 cm^-1^ on the heat maps correlates well with the size of PTFE which was embedded underneath the skull (5 mm × 5 mm). It is noted, however, that spectral signal from PTFE is observed outside of its 5 mm × 5 mm area. Unlike confocal Raman microscopy techniques which are useful for generating high spatial information, in part through the use of a focused beam, SORS utilizes a diffuse beam and the concept of photon migration in turbid media. Thus, when the scattered Raman photons are eventually returned to the collection region, they will have undergone multiple scattering events and will therefore contain some spectral information related to the analyte obscured by the barrier, e.g. PTFE, even if the analyte is not present in that region. It is reasoned that this is therefore why a spectral contribution of PTFE is observed outside the 5 × 5 mm area. Nonetheless, clear distinction of areas where the PTFE is present and where it is not is displayed on the heat maps. Furthermore, it is noted that as the spatial offset is increased, e.g. from 2 to 3 mm, a reduction in the overall spectral intensity was obtained. This observation is not exclusive to regions related to PTFE but also regions related to tissue and bone and is typical of SORS spectra collected at a larger offset due to photon migration losses. As such, a compromise must be made between obtaining SORS spectra that contains spectral information predominantly relating to the analyte with spectra that generate good signal-to-noise ratios. Thus, it was reasoned that a spatial offset of 2.5 mm would be suitable for *in vivo* imaging of GBM using SESORS, since it facilitated suppression of the surface signal, i.e. tissue (1440 cm^-1^), diminished the spectral contribution from bone (957 cm^-1^), and in tandem, generated a strong contribution from PTFE (739 cm^-1^) in the acquired signal (**Figure 3 and 4)**. Furthermore, good signal-to-noise ratios were also observed at a 2.5 mm spatial offset meaning that the resulting spectra had well defined and easily interpretable spectral features with significant contribution from the analyte.

**Figure 5:**
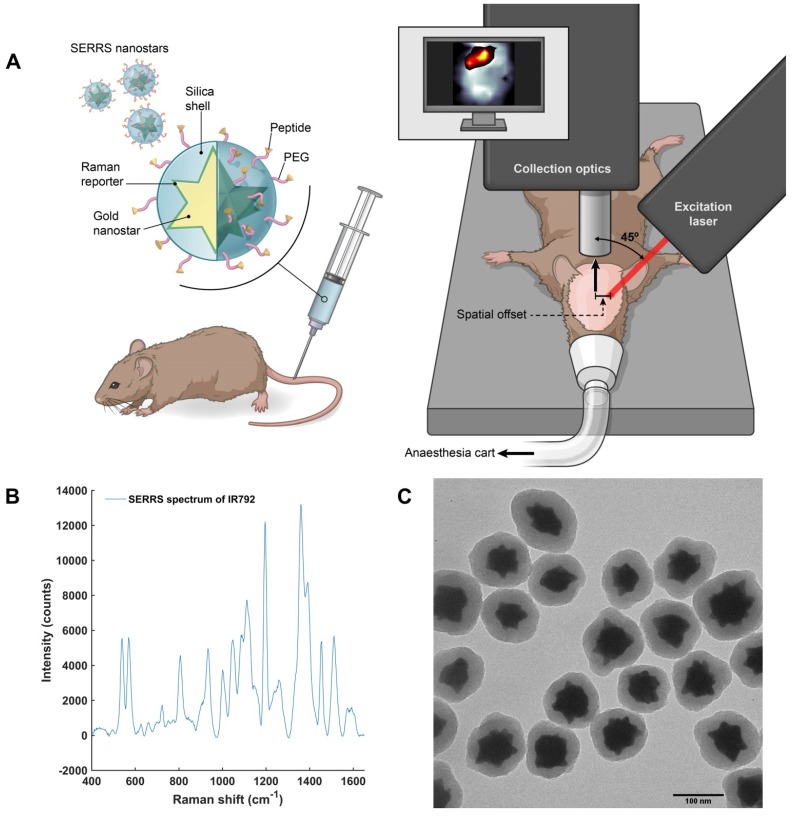
** Concept and characterization of integrin-targeted SERRS-Nanoprobes for in vivo imaging of GBM using SESORRS**. (a) Conceptual figure outlining integrin-based detection of GBM through the use of cRGDyK-conjugated SERRS nanostars. The dimensions and spatial offset are not drawn to scale. Gold nanostars were functionalized with a Raman reporter which generates a unique spectrum described as a “Raman fingerprint”. The SERRS nanostars were then encapsulated in a thin silica shell which was subsequently functionalized with cRGDyK to develop SERRS nanostars capable of targeting integrins overexpressed in GBMs. Following injection of integrin-targeting nanoparticles, in vivo SESORRS imaging of GBM was performed using a custom-built SORS system. The unique fingerprint of the Raman reporter spectrum was tracked non-invasively through the skull using the SESORRS approach. (b) SERRS spectra of the nanostars aquired with conventional Raman, averaged from 3 samples, 5 accumulations, 785 nm, 20mW, 10 ms integration, 5 acquisitions. (c) TEM image of silicated, RGD-coated gold nanostars with an average diameter of 120+/-9 nm and a thin silica shell of 23 ± 4 nm.

After these characterization and optimization experiments, we then proceeded to *in vivo* studies (concept illustrated in **Figure 5a)**. In order to both target and image GBM *in vivo* using SESORS, integrin-targeted SERRS NPs were synthesised by conjugating cyclic-RGDyK to silica coated SERRS active gold nanostars. This created SERRS contrast agents which could specifically target GBM and, in turn, be used as tracking agents *in vivo*. The commercially available near-IR active dye (IR792) was selected for this study since it has an electronic transition that corresponds to that of the laser wavelength, meaning it would generate an enhancement in Raman signal when propagated with incident laser light (785 nm). The SERRS nanotags were therefore tracked using "surface-enhanced spatially offset resonance Raman spectroscopy" (SESORRS). The SERRS spectrum of the integrin-targeting NPs is shown in **Figure 5b**. The chemical structure of dye IR792 perchlorate is shown in the supporting information (**Figure S4**). SERRS NPs were characterized using conventional Raman spectroscopy (CR), dynamic light scattering and zeta potential analysis (**Table S1**, supporting information) as well as transmission electron microscopy (TEM), (**Figure 5c**). The gold nanostars had an average diameter of 120 nm and were coated with a silica shell with an average thickness of 23 nm.

To target GBM *in vivo*, a 100 µL (8 nM) dose of RGD-SERRS NPs was injected via the tail vein of RCAS-*Pdgfb* / N-tva GBM bearing mice (n=5) (**Figure 5a**). All mice had left frontal tumors, confirmed by MRI at 4 weeks post DF-1 cell injection. After 18-24 hours, the mice were anesthetized using isoflurane inhalation for imaging studies using SESORRS and conventional Raman. Thus, all imaging experiments were carried out on live mice. Each mouse was then transferred to a translational *xy* stage (**Figure 5a**), and fixed in position using surgical tape. The head of the mouse was positioned under the SORS set-up. SESORRS images were then acquired by moving the mouse in the *x* and *y* direction, in steps of 1 mm, to create an image of 12 × 12 pixels. The same area was then mapped using conventional Raman. For SESORRS imaging, a laser power of 130 mW was used. The incident beam was elliptical with a shorter radius of 1.5 mm and a longer of 2 mm. Therefore, the power density equates to an average of 13.8 mW/mm^2^. This laser power compares well with laser illumination intensities used in other SORS studies related to biomedical applications which also employ large spot sizes (diameter ~4 mm 40, 41. Although the power density/mm^2^ is approximately four times higher than that of the safety limit associated with accidental laser exposure to skin (4 mW/mm^2^), this system is expected to fall within the permissible exposure limits associated with class 1C lasers 41. Class 1C lasers were newly established in IEC 60825 version 07-2015 and cover laser systems that are designed to be in direct contact, or in close proximity to the subject, e.g. skin 63. In addition, the power levels here are significantly lower than the laser illumination levels used in a study by Enejder *et al*., where the authors successfully detected glucose in a non-invasive and quantitative manner using power levels of 300 mW/mm^2^ in 17 human subjects 64. It should also be noted that maximum permissible exposure limits (MPE) have not been established for preclinical models 65, however the power density/mm^2^ used in this study is significantly lower in comparison to previous studies involving the detection of SERRS NPs using conventional Raman techniques 66-68.

Following SESORRS imaging, each mouse was then imaged using CR serving as a direct comparison and control. In CR, the excitation light was delivered and collected through the same probe at a 180˚ angle to the mouse's head, i.e. a 180˚ back-scattering configuration. The probe was focused in an attempt to see beneath the skull. The spot size of the laser beam was set to 500 µm. Since one of the key benefits of the SORS technique is its ability to generate Raman spectra using such low power densities through the deployment of an expanded beam, the power density in the CR control studies was therefore normalised to that used in SORS measurements. This meant that a 2.7 mW laser power should have been used for CR experiments. However, when this power was used, no spectral information on the SERRS NPs, tissue or skull was generated. Therefore, a 20 mW laser power was used for control experiments. This power was chosen as it was the lowest power on our system which generated spectral information that corresponded to either that of the SERRS nanotags or biological tissue, e.g. the skull. This therefore gave a power density of 102 mW/mm^2^, which was over five times higher than that used for the SESORRS measurements. No apparent skin damage was noted after imaging using either SORS or CR techniques and all mice regained normal behaviour post-imaging.

**Figure 6:**
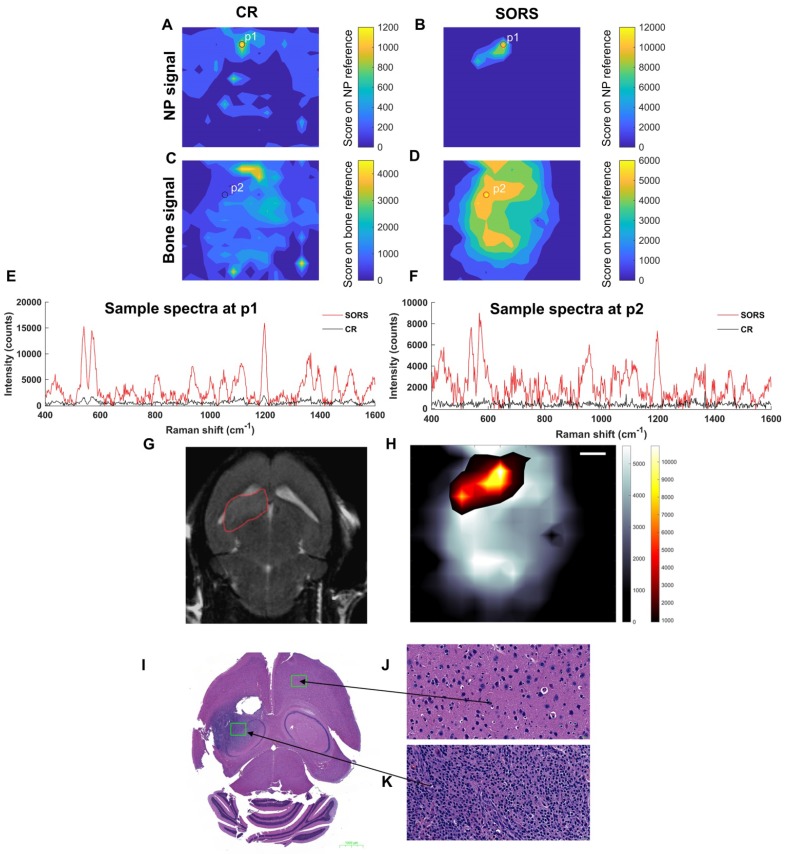
Detection of GBM in mice (n=5) using SESORRS and conventional Raman (non-SORS; CR for short) methods. For all heat maps, a CLS fit was applied to both the SORS and CR spectra. (a) The detection and CLS contribution of SERRS NPs through the skull using a CR imaging approach. (b) The detection and CLS contribution of SERRS NPs through the skull using a SORS imaging approach. (c) The detection and CLS contribution of bone through the skull using a CR imaging approach. (d) The detection and CLS contribution of bone through the skull using a SORS imaging approach. (e) A spectrum collected at the point of maximum intensity (P1) that relates to the greatest CLS contribution from the SERRS NPs imaged using SORS (red) and CR (black), respectively. SORS signal is an order of magnitude higher than that collected using CR. (f) A spectrum collected at the point where the greatest CLS contribution of bone is observed (P2) and a comparison spectrum collected from the same point using CR (black). (g) 2D axial T2-weighted MRI taken 4 weeks post injection of DF-1 cells confirms presence of a left frontal tumor (outlined in red). Images were acquired using a slice thickness of 0.7 mm taken at a depth of 3.6 mm. The orientation of the original MRI image was inverted horizontally so that left corresponds to left in the image, in contrast to the inverted radiological convention. (h) The SORS heatmap (shown in a) was superimposed onto the SORS bone heat map (shown in b). The SORS image delineates the tumor margin in good agreement with the MRI. (i) H&E stained 5 µM section of the brain. The arrows represent the areas of the slice which represent healthy tissue (j) and cancerous tissue (k), thus further corroborating the SESORRS imaging data. Images in (j) and (k) were taken at 40× magnification. SORS measurements were acquired using a power density of 13.8 mW/mm^2^, 2.5 mm spatial offset, 3s integration time, 5 acquisitions, 785 nm excitation wavelength. CR measurements were acquired using a power density of 102 mW/mm^2^ (> 5× that of SORS), 3s integration time, 5 acquisitions, 785 nm excitation wavelength.

**Figure 6** describes the non-invasive *in vivo* imaging of integrin-targeting SERRS nanoparticles through the skull in GBM bearing mice by means of CR and SORS. A least squares fit was applied to data collected using either the CR (**a, c**) or the SORS approach (**b, d**). The heatmaps provide information on spectra that correspond to that of the SERRS nanoparticle (**a, b**) and that of bone (**c, d**). In the heatmaps obtained using SORS (**b, d**), stronger Raman signal intensities were acquired (despite the 5× lower power density) and, in addition, higher tumor-to-background contrast is observed in comparison to those collected using CR (**a, c**). Representative spectra collected at position 1 (P1) and position 2 (P2) by means of CR or SORS approaches are also shown (**e**,** f**). P1 and P2 were chosen based on the CLS contribution of either the SERRS-NPs or bone from the SORS least squares maps (**b**, **d**). In both instances, the data demonstrate clearly that spectra acquired using the SORS technique generate higher signal intensities and display clear spectral information which corresponds to the Raman signatures of the SERRS NPs, e.g. the peak at 1198 cm^-1^ and bone, e.g. 957 cm^-1^. This is in contrast to Raman spectra obtained using CR, which, despite displaying a corresponding SERRS NP peak at 1198 cm^-1^ (**e**), were typically associated with low intensity values and poorer signal to noise. In order to delineate the tumor margins, the corresponding SORS CLS heatmaps of the SERRS NPs (**b**) were then superimposed on SORS heatmaps of bone (**d**) to create images such as in (**h**). The area of heat intensity which represents the SERRS NPs was shown to be in accordance with the tumor region observed in the MRI scan, indicating successful detection of GBM *in vivo* using SESORRS. *Ex vivo* H&E staining confirmed the presence of healthy brain tissue in SERRS NP signal negative areas (**j**) and the presence of tumor tissue in the SERRS NP signal positive area (**k**). The histopathological findings were concordant with the MRI data.

The main aim of this study was to enable visualization of GBMs non-invasively using SESORRS. This was achieved through utilization of integrin-targeted SERRS NPs to specifically target the tumor. We used the genetically engineered RCAS-Pdgfb-driven/tva murine GBM model because this tumor model is considered to closely resemble the development and biology of GBMs in humans, thus adding further to the clinical potential of our results 53. Previous work from our group has demonstrated the successful targeting of GBM using cyclic-RGD as a targeting moiety, thus the purpose of this work was not to investigate potential targeting ligands, but to demonstrate the potential of SORS over CR techniques for the non-invasive *in vivo* imaging of cancerous tissue using SERRS NPs. The results presented here clearly demonstrate that through utilization of the SORS approach, it is possible to generate strong and distinct spectra that correspond to images obtained using MRI. The results also display high contrast and high signal specificity for the area of interest, i.e. the tumor region. It is reasoned that it would have been possible to image the integrin-targeted SERRS NPs using CR methods, however this would have certainly required the use of higher power densities (mW/mm^2^). Furthermore, CR of biological tissue is often associated with a high fluorescent background, thus potentially masking important Raman signatures of interest 30. This interference was largely suppressed through application of the SORS technique. As such, through application of SORS in combination with a diffuse beam, it is possible to gain excellent spatial information regarding tumor location using an extremely low power density (over five times less than that used for CR measurements). It is well established that the choice of laser power is an extremely important factor to consider in Raman spectroscopy applications, and often high laser powers are utilized to obtain Raman spectra that provide qualitative and quantitative information.

Therefore, if SERS imaging is going to be translated into the clinics, researchers must consider the effects and overall acceptance by clinical governing bodies regarding the use of high laser powers, specifically power density, for *in vivo* Raman imaging applications. We believe the method presented here, i.e. the combination of SORS and SERRS, provides a means to reducing the high power density necessary for CR imaging approaches, whilst at the same time, allowing the imaging of deeper-seated tissues. However, it is accepted that such clinical translation would also require the approval of SERRS NPs for use in humans by regulatory bodies such as the FDA.

In this instance a spatial offset of 2.5 mm was used for SORS imaging, however it is reasonable to assume that the other spatial offsets would have been suitable for SESORS imaging, namely the 2 and 3 mm spatial offset investigated using the PTFE-tumor phantoms. Nevertheless, this was a proof of concept study which aimed to introduce the application of SESORS for *in vivo* imaging, thus the main aim was not to evaluate the most suitable offset, but to demonstrate the potential applicability and suitability of SESORS as an overall imaging technique. It is worth emphasising that all mice were alive during imaging by both SORS and CR and that they regained consciousness post-imaging. Biodistribution studies on the fate of the SERRS NPs yielded expected results, revealing the accumulation of NPs in the liver, spleen and lymph nodes (**Figure S3**). One limitation associated with this study was the lack of a white light image to relate the acquired Raman maps with a physical point on the mouse anatomy. This issue was overcome through the use of MRI scans generated prior to Raman imaging, however it is recommended that this be incorporated in future studies for fast correlation between Raman maps and the subject under study. Future work will seek to generate the spectral information on the whole tumor, i.e. not just in *x* and *y* but also in the *z* direction using a SESO(R)RS approach.

## Conclusion

This proof of concept study demonstrates the capability and advantages of the SORS technique in combination with SERRS NPs, for *in vivo* imaging applications. To the best of our knowledge, this is the first report of *in vivo* SESORS imaging. In contrast to CR, which is associated with high power densities and is therefore less favourable for clinical translation, our results demonstrate the successful detection, and more importantly profiling, of GBM* in vivo* using a SESORRS approach. It is reasoned that the use of SESORS for *in vivo* imaging will not be exclusive to brain cancer imaging but could also be applicable to the monitoring of a wide range of diseases provided the SERRS NP can actively target the region of interest. Based on this, we expect to see a transition towards the application of SORS-based imaging strategies for preclinical imaging over conventional Raman approaches. As such, this novel work represents a significant step forward in the detection of vibrational fingerprints located at depth *in vivo* and represents an important step forward in the use of SESORS for potential clinical applications, particularly in the realm of cancer imaging.

## Supplementary Material

Supplementary figures and tables.
