# Supplementary material for: Non-invasive In Vivo Imaging of Cancer Using Surface-Enhanced Spatially Offset Raman Spectroscopy (SESORS)
Source: Theranostics. 2019 Aug 13;9(20):5899–913. doi: 10.7150/thno.36321 (PMC6735365; doi:10.7150/thno.36321)
Supplement: Supplementary file 1 — Supplementary figures and tables. [file thnov09p5899s1.pdf]

Supporting information

Non-invasive In Vivo Imaging of Cancer Using Surface-Enhanced Spatially Offset Raman Spectroscopy (SESORS)

Authors

Fay Nicolson[1], Bohdan Andreiuk[1], Chrysafis Andreou[2], Hsiao-Ting Hsu,[1], Scott Rudder[3] and Moritz F. Kircher*[1,4,5]

Affiliations

1. Department of Radiology, Memorial Sloan Kettering Cancer Center, New York, New York 10065, United States
2. Department of Electrical and Computer Engineering, University of Cyprus, Nicosia, Cyprus
3. Innovative Photonic Solutions, Monmouth Junction, New Jersey 08852, United States
4. Molecular Pharmacology Program, Sloan-Kettering Institute, New York, New York 10065, United States
5. Department of Imaging, Dana-Farber Cancer Institute & Harvard Medical School, Boston, Massachusetts, 02215, United States

Corresponding author:
Moritz F. Kircher, Department of Imaging, Dana-Farber Cancer Institute & Harvard Medical School, Boston, Massachusetts 02215, United States

E-mail: moritz_kircher@dfci.harvard.edu

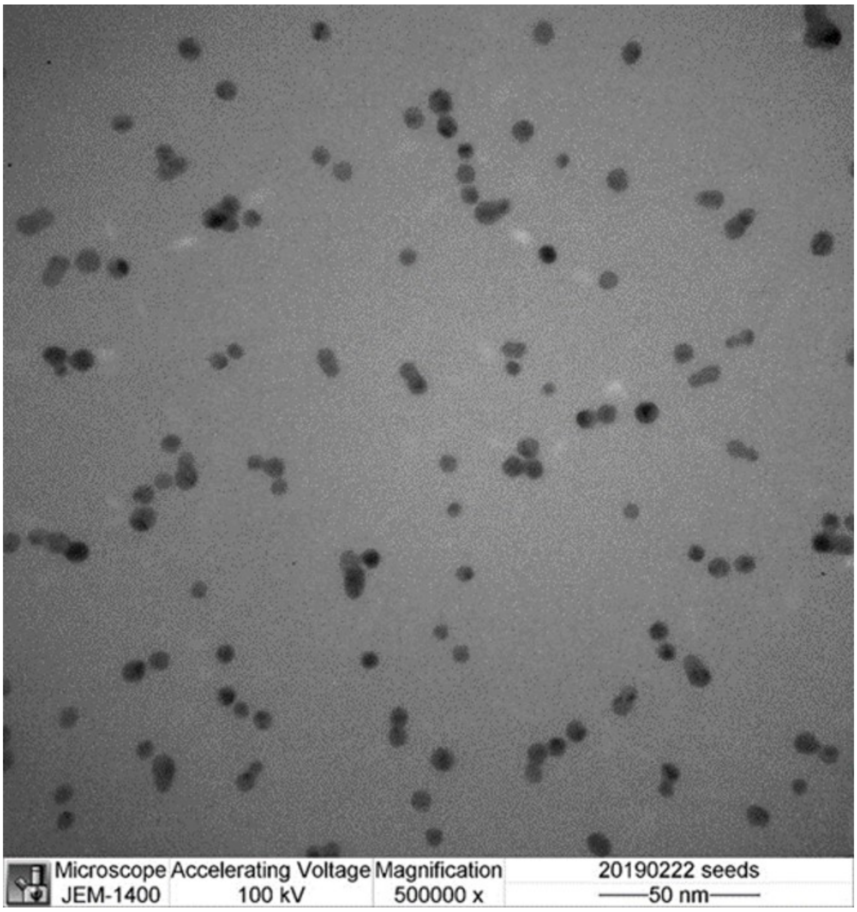

Figure S1. Transmission electron microscopy (TEM) image of 5 nm gold seeds used for the nanostar synthesis. TEM was performed on a JEOL JEM 1400 Transmission Electron Microscope at 100 kV.

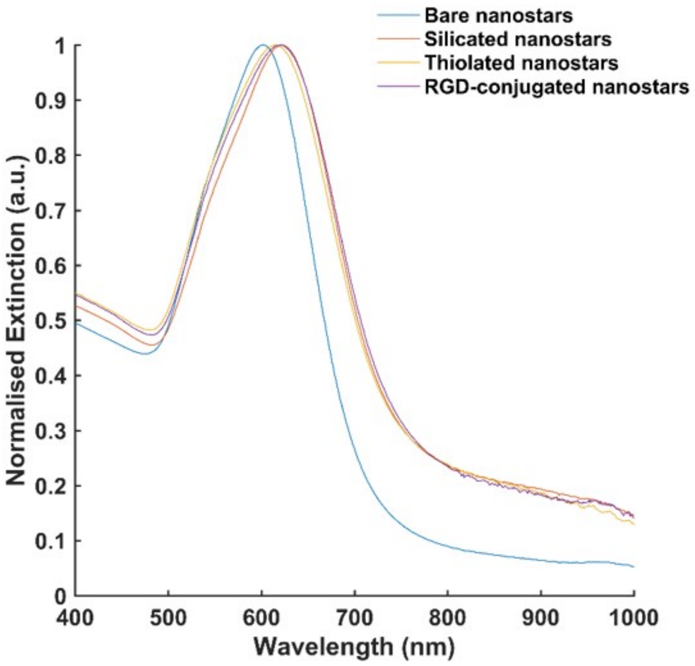

Figure S2. Normalized extinction spectroscopy on bare nanostars, silicated nanostars, thiolated nanostars and RGD-conjugated nanostars. The λmax values were 602, 622, 615 and 621 nm respectively. A SpectraMax ID 5 plate reader (Molecular Devices) was used for optical characterization of the nanoparticles.

Surface modification of the silica shell

Surface modification of silica-coated nanostars with thiols was reflected in the change of the zeta potential of the nanoparticles. It decreased from -39 to -50 mW due to higher acidity of surface -SH groups compared to surface -OH groups (Table S1). After surface -SH groups reacted with PEG-cRGDyK through thiol-maleimide coupling, the zeta potential became -38 mW, reflecting the disappearance of -SH groups from the surface. As expected, no significant changes in hydrodynamic diameter of the silicated nanostars occurred during surface modification (Table S1).

Table S1. Physical properties of gold nanostars at various stages of peptide conjugation.

	**Zeta potential (mV)**	**Standard Deviation (mV)**	**Hydrodynamic size (Z-average) (nm)**	**Standard Deviation (nm)**
**Silicated**	-39	1	142	1
**Thiolated**	-50	1	141	2
**RGD-conjugated**	-38	2	146	3

Biodistribution studies

Biodistribution analysis was performed to determine the fate of the SERRS nanotags *in vivo*. RCAS-Pdgfb/N-tva GBM-bearing mice (n=5) were injected with 8 nM of SERRS nanostars (100 µL). Following imaging using SESORS and conventional Raman approximately 24 hours later, mice were euthanized using CO_2 asphyxiation. For each mouse (n = 5), organs were harvested, weighed, and homogenized. Tissue homogenates were placed in 384-well plates. Raman images of the plates were acquired using 10% laser power (785 nm), 1 s acquisition, 5× objective, 250 µM step size (Renishaw InVia, Hoffman Estates, IL). As expected, the results show clear uptake of SERRS nanostars in liver and spleen and lymph nodes (**Figure S3**).

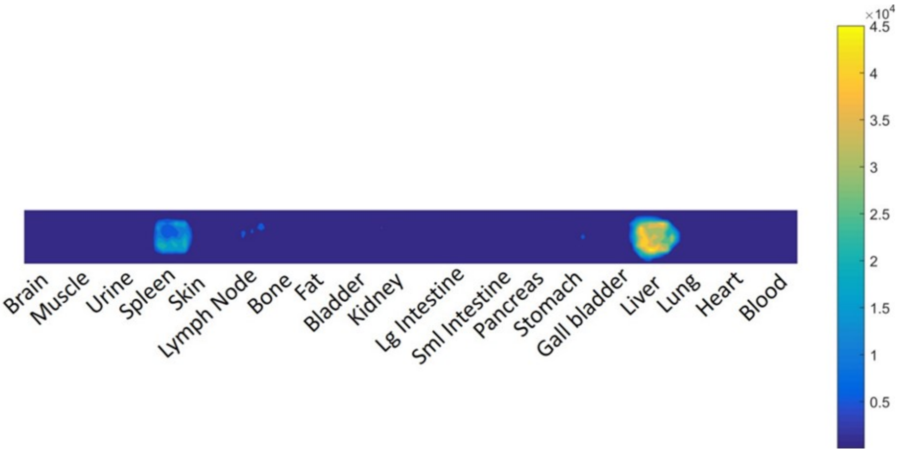

Figure S3. Tissue distribution of SERRS nanostars. RCAS-Pdgfb/N-tva GBM-bearing mice (n=5) were injected with 100 µL of 8 nM of SERRS nanostars. Following imaging with SESORS and CR approximately 24 hours later, mice were asphyxiated using CO_2 and their organs harvested. The organs were then analysed by Raman imaging (10% laser power, 1 s acquisition time, 5× objective) to determine the relative accumulation in different tissues. Images are representative of n = 5 mice, 1 organ per well.

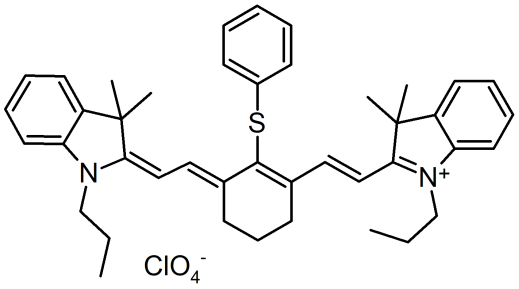

Figure S4. Chemical structure of dye IR-792 perchlorate
